# Activation of calpain-1 in human carotid artery atherosclerotic lesions

**DOI:** 10.1186/1471-2261-9-26

**Published:** 2009-06-18

**Authors:** Isabel Gonçalves, Mihaela Nitulescu, Takaomi C Saido, Nuno Dias, Luis M Pedro, José Fernandes e Fernandes, Mikko PS Ares, Isabella Pörn-Ares

**Affiliations:** 1 Department of Clinical Sciences, Lund University, Malmö, Sweden; 2 Department of Cardiology and Internal Medicine, Lund University, Malmö, Sweden; 3 Laboratory for Proteolytic Neuroscience, RIKEN Brain Science Institute, 2-1 Hirosawa, Wako-shi Saitama 351-0198, Japan; 4 Department of Vascular Diseases Malmö-Lund, Lund University, Malmö, Sweden; 5 Instituto Cardiovascular de Lisboa, Lisbon, Portugal; 6 Department of Laboratory Medicine/Experimental Pathology, Lund University, Malmö, Sweden; 7 Research Program of Molecular Neurology, Institute of Biomedicine/Biochemistry, University of Helsinki, Helsinki, Finland

## Abstract

**Background:**

In a previous study, we observed that oxidized low-density lipoprotein-induced death of endothelial cells was calpain-1-dependent. The purpose of the present paper was to study the possible activation of calpain in human carotid plaques, and to compare calpain activity in the plaques from symptomatic patients with those obtained from patients without symptoms.

**Methods:**

Human atherosclerotic carotid plaques (n = 29, 12 associated with symptoms) were removed by endarterectomy. Calpain activity and apoptosis were detected by performing immunohistochemical analysis and TUNEL assay on human carotid plaque sections. An antibody specific for calpain-proteolyzed α-fodrin was used on western blots.

**Results:**

We found that calpain was activated in all the plaques and calpain activity colocalized with apoptotic cell death. Our observation of autoproteolytic cleavage of the 80 kDa subunit of calpain-1 provided further evidence for enzyme activity in the plaque samples. When calpain activity was quantified, we found that plaques from symptomatic patients displayed significantly lower calpain activity compared with asymptomatic plaques.

**Conclusion:**

These novel results suggest that calpain-1 is commonly active in carotid artery atherosclerotic plaques, and that calpain activity is colocalized with cell death and inversely associated with symptoms.

## Background

Calpains are calcium-dependent cysteine proteases that are known to be involved in the proteolysis of a number of proteins during mitosis and cell death [1,2]. The calpains constitute a large family of distinct isozymes that differ in structure and distribution [3], and three members of this family are ubiquitous – calpain-1 (μ-calpain), calpain-2 (m-calpain), and calpain-10. A study with embryonic fibroblasts from mice with genetically disrupted *capn4*, which codes for the regulatory subunit of both calpain-1 and -2, showed that calpains are required for activation of caspase-12 and c-Jun N-terminal kinase in ER-stress-induced apoptosis [4]. The specific endogenous protein inhibitor calpastatin, which modulates calpain activity *in vivo*, is cleaved during apoptosis [5]. The cytoskeletal protein α-fodrin is another death substrate that may be cleaved by calpains or caspases [1,6]. Additional calpain substrates known to be involved in apoptosis are Bax [7], Bid [8], p53 [9], and procaspase-3, -7, -8, and -9 [10,11]. In a previous study, we found that oxidized low-density lipoprotein (oxLDL)-induced death of human microvascular endothelial cells (HMEC-1) was accompanied by activation of calpain-1 [12]. The calpain-1 inhibitor, PD 151746, decreased oxLDL-induced cytotoxicity, and the 80 kDa subunit of calpain-1 was autoproteolytically cleaved in oxLDL-treated HMEC-1 cells, indicating that the enzyme was activated. The Bcl-2 protein Bid was also cleaved during oxLDL-elicited cell death, and this was prevented by calpain inhibitors, but not by inhibitors of cathepsin B or caspases.

Vascular calcification is present in 80% of significant atherosclerotic lesions and in at least 90% of patients with coronary artery disease [13]. Calcification can apparently begin at any point of plaque formation and seems to be a complex mechanism [14]. Since vascular calcification has been shown to correlate with elevated serum calcium [15], and oxLDL plays a central role in atherogenesis [16], we hypothesized that calpains may be activated in atherosclerotic lesions. Therefore, the primary aim of the present study was to analyze atherosclerotic plaques for possible calpain activity.

## Methods

### Materials

Anti-calpain-1 large subunit monoclonal Ab was from Chemicon International (Temecula, CA, MAB3082), anti-α-tubulin monoclonal Ab was from Oncogene Research Products (Boston, MA, #CP06). HRP-coupled goat anti-rabbit and goat anti-mouse immunoglobulins were from Dako A/S (Glostrup, Denmark). Reagents not listed here were obtained from Sigma, unless otherwise stated in the text.

### Atherosclerotic plaques

Twenty-nine human atherosclerotic carotid plaques, from 26 patients (67 ± 8 years old, 21 males), were removed *en bloc *by carotid endarterectomy by one surgeon. Twelve plaques were associated with ipsilateral hemispheric symptoms in the last month and 17 were not associated to any symptoms after neurologic evaluation. Cardiovascular risk factors such as hypertension (systolic blood pressure > 140 mmHg), diabetes, coronary artery disease, tobacco use (in the past or current) and dyslipidemia were recorded, as well as the medication of these patients. Patients with atrial fibrillation, aortic valve disease, mechanic heart valves, ipsilateral carotid artery occlusion or restenosis after previous carotid endarterectomy were excluded. Informed consent was given by each patient. The study was approved by the local ethical committee. The histological characteristics of symptomatic and asymptomatic plaque samples have been published previously [17]. In short, carotid plaques from symptomatic patients have shown lower levels of hydroxyapatite, higher levels of elastin, cholesterol esters, unesterified cholesterol, triglycerides, more cells, DNA, and soluble protein [18] compared to those from asymptomatic patients.

### Sample Preparation

The plaques removed by endarterectomy were cleaned with isotonic NaCl containing heparin (5 U/ml), to avoid blood contamination, and thereafter the plaques were immediately snap frozen in liquid nitrogen. Two-mm-thick fragments from the stenotic region of the frozen plaques were removed for histology. Plaques were weighed, cut into pieces while still frozen, and homogenized as previously described [19]. An aliquot was taken from each plaque for western blot analysis, and protein content was analyzed by the method of Lowry.

### Immunoblotting and calpain activity

Loading buffer (final concentrations: 50 mmol/L Tris-HCl [pH 6,8], 2% SDS, 10% glycerol, 0,1% bromophenol blue, and 30 mmol/L dithiothreitol) was added to homogenized samples, and they were heated to 90°C in a heating block for 5 min. Proteins were separated under reducing conditions in SDS-polyacrylamide gels and then Western blotted onto PVDF filters. Blots were blocked with Tris-buffered saline containing 5% dry milk powder, and then incubated for 1–2 h with anti-proteolyzed 150 kDa α-fodrin pAb [20](diluted 1:200), anti-α-tubulin mAb (1:500), or anti-calpain-1 mAb (1:2000). The blots were subsequently incubated with a peroxidase-conjugated secondary Ab, and bound Ab was assayed by enhanced chemiluminescence detection (Santa Cruz Biotechnology, Santa Cruz, CA). To estimate the level of calpain activity, we performed densitometric analysis of Western blots with a Fluor-S MultiImager (Bio-Rad, Rockford, IL). The optical density of 150 kDa α-fodrin bands and tubulin bands was scanned, and the calculated ratio (OD_α-fodrin_/OD_tubulin_) for each plaque sample was used in statistical analysis.

### Immunohistochemistry

Two-millimeter-thick fragments from the stenotic regions of the frozen plaques were embedded in O.C.T. compound (Tissue-Tek, Sakura), cryo-sectioned in serial 8-μm sections, and mounted on coated slides. Tissue sections for immunohistochemistry were fixed with 4% paraformaldehyde in phosphate buffer. Membranes were permeabilized in 0.5% Triton X-100. Endogenous peroxidase activity was quenched by incubating sections for 5 min in 0.9% H_2_O_2_. Thereafter sections were blocked with 10% goat serum in PBS for 30 min. Primary antibody, rabbit anti-cleaved-α-fodrin (150 kDa; ref. [20]), was diluted 1:200 and incubated overnight at 4°C in a humidified chamber. Sections were incubated with biotinylated secondary Ab (goat anti-rabbit, Vector Laboratories, Burlingame, CA) at a dilution of 1:200 for 60 min. Thereafter, sections were incubated with peroxidase- or alkaline phosphatase-labeled Streptavidin (for brown or blue stain, respectively; Vectastain ABC-AP kit, Vector Laboratories). In the case of double-staining, TUNEL was performed after the anti-cleaved-α-fodrin staining. Sections were developed with diaminobenzidine (Vector Laboratories), and counterstained with hematoxylin. Negative controls included substitution of the primary Ab with phosphate buffer.

For TUNEL staining, consecutive tissue sections were fixed with 4% paraformaldehyde in phosphate buffer and stained for apoptosis, using TUNEL *In Situ *Cell Death detection kit POD (Roche Applied Science, Indianapolis, USA), according to the manufacturer's instructions. Samples were viewed with an Olympus BX60 microscope and photographed.

### Statistical Analysis

Results were normalized to the wet weight of the plaques. We used χ^2 ^analyses to investigate associations with dichotomous variables. Two-group comparisons were performed with the use of the Mann-Whitney non-parametric test. Spearman's rho was used for correlation analyses. Statistical analysis was performed with the use of SPSS 12.0 for Windows.

## Results

### Calpain activity and apoptosis

**Figure 1:**
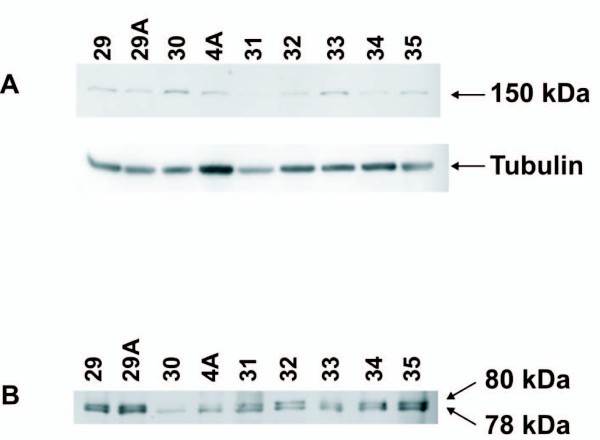
**Calpain-1 is activated in atherosclerotic plaques and cleaves α-fodrin**. A, Plaque homogenates were processed for Western blotting, and the PVDF membrane was probed with an anti-proteolyzed α-fodrin Ab specific for the 150-kDa fragment produced by calpain activity. The membrane was subsequently stripped and re-probed with anti-α-tubulin Ab as a loading control. B, same as A, except that the PVDF membrane was probed with anti-calpain-1 large subunit Ab. The blots show 9 samples (the id number of each plaque is depicted above the lanes) of 29 analyzed. All 29 samples contained the 150 kDa α-fodrin fragment as well as the autolytic fragment of calpain-1.

Use of Western blot analysis and a specific antibody to detect a calpain-proteolyzed α-fodrin fragment has proven to be one of the most reliable methods to demonstrate calpain activation in cell lysates [6,20]. We employed a polyclonal antibody specific for the calpain-proteolyzed 150-kDa α-fodrin fragment [20] to determine whether calpain activation occurs in atherosclerotic plaques. We found that all plaque samples contained the calpain-generated 150-kDa α-fodrin breakdown product (Figure 1A). These results show that calpain was activated and catalysed the cleavage of α-fodrin in the atherosclerotic plaques.

The autoproteolytic cleavage of the 80 kDa subunit of calpain-1 and -2 is known to be associated with activation of these enzymes [3]. To further verify the activation of calpain in atherosclerotic plaques, we used a monoclonal antibody against the 80 kDa subunit of calpain-1 on western blots, and we observed the 78 kDa autoproteolysis product of calpain-1 in all samples (Figure 1B). The detection of cleaved calpain-1 provided further evidence for active calpain in atherosclerotic plaques.

**Figure 2:**
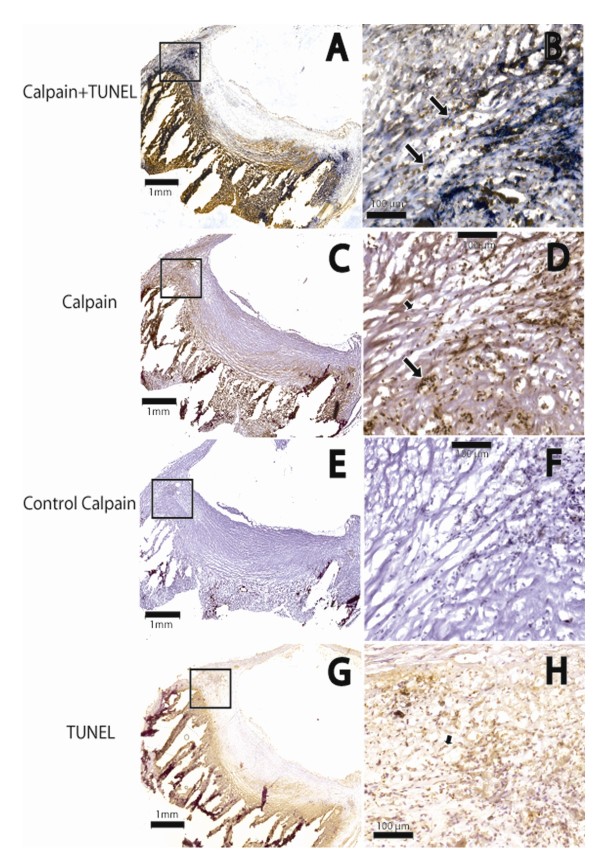
**Immunohistochemical stainings of a human atherosclerotic plaque**. The region inside the square in A, C, E, and G is amplified in B, D, F, and H, respectively. A and B, sections were double stained for calpain (proteolyzed α-fodrin, in blue) and TUNEL (in brown), showing colocalization of these two stainings. The arrows in B show cells staining positively for calpain and TUNEL. C and D, sections were incubated with anti-proteolyzed α-fodrin antibody. α-fodrin fragments, resulting from the presence of active calpain, are present in the core and shoulder regions of the plaques and in some scattered areas of the fibrous cap. This is also seen in A with the blue colour. In D, cells stained brown are positive for calpain activity (long arrow), whereas non-stained cells (short arrow) are not. E and F, negative control (primary antibody omitted). G and H, TUNEL staining for cell death (in brown). The arrow in H points to a dying cell, staining brown.

The preparation of homogenates for western blot analysis from plaque samples is a lengthy process, and despite the included protease inhibitors it could be argued that the proteolysis detected in Figure 1 might be artifacts from the processing of the samples. Therefore we performed immunohistochemistry on sections of the plaque samples, and the results from this analysis verified that calpain was indeed activated in the plaques. Figure 2 shows a plaque with immunohistochemical staining of the calpain-generated 150-kDa α-fodrin fragment, as well as apoptotic cell death detected by TUNEL staining. Interestingly, calpain activity (Figure 2A, B, C, and 2D) colocalized with cell death, as shown in Figure 2A, B, G, and 2H.

### Calpain activity and plaque characteristics

**Figure 3:**
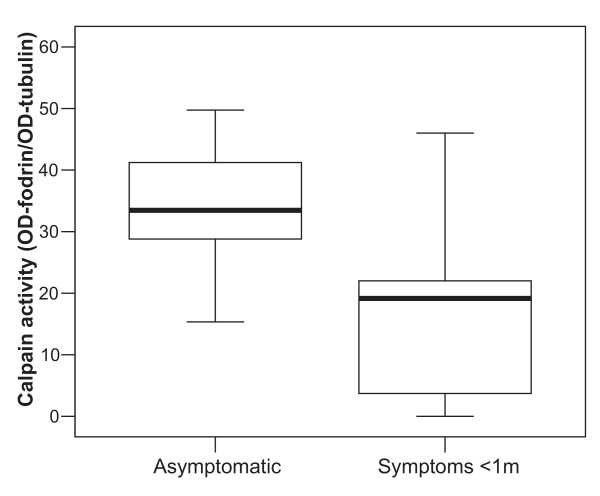
**Differences in calpain activity of carotid plaques from asymptomatic and symptomatic patients**. Calpain activity was estimated as described in Materials and Methods. The difference between asymptomatic and symptomatic plaques was statistically significant (p = 0.034). The box plot shows minimum, first quartile, median, third quartile, and maximum levels.

When calpain activities were quantified from western blots, we found that symptomatic carotid plaques displayed significantly lower calpain activity (on average 38.0% less) compared with plaques not associated with symptoms (Figure 3). Since calpain-dependent apoptosis has been detected in simvastatin-treated rat vascular smooth muscle cells [21], we decided to perform statistical analyses comparing calpain activity of the plaques and statin intake. However, we found no significant difference in calpain activity between untreated patients and those taking statins (data not shown). There were no significant differences in calpain activity between untreated patients and those taking anti-hypertensives (including calcium-channel blockers; Tables 1 and 2). Neither was there any significant association found between calpain activity and the registered cardiovascular risk factors.

**Table 1:** Most relevant clinical characteristics of the symptomatic patients.

**Symptomatic (n = 12)**	**No**	**Yes**
Diabetes	9	3
Hypertension	4	8
Heart disease	5	7
Smoking	4	current 3 and ex 5
Obesity	12	0
Family history of cardiovascular disease	11	1
Peripheral arterial disease	11	1
Statin	10	2
Anti-hypertensives	5	7 (3 CCB)

CCB, calcium channel blockers

**Table 2:** Most relevant clinical characteristics of the asymptomatic patients.

**Asymptomatic (n = 17)**	**No**	**Yes**
Diabetes	13	4
Hypertension	5	12
Heart disease	6	11
Smoking	9	current 6 and ex 2
Obesity	13	4
Family history of cardiovascular disease	17	0
Peripheral arterial disease	10	7
Statin	13	4
Anti-hypertensives	6	11 (5 CCB)

CCB, calcium channel blockers

## Discussion

Our present data demonstrate unequivocally that calpain was activated in atherosclerotic plaques and that calpain activity was co-localized with cell death. Interestingly, in a previous study on these carotid plaques, those associated with symptoms had 70% lower amounts of calcium (hydroxyapatite) [19]. This is in accordance with other studies suggesting that calcium could make plaques more stable [22], limiting the spread of inflammation [14]. A calcified nodule within or close to the plaque cap can protrude and lead to rupture [23]. However, if the calcified areas coalesce, the interfaces between rigid and distensible areas as well as the mechanical stress decrease [14]. Therefore, depending on their topography in the lesion, calcified areas can function as a protective "shell".

The presence of bone proteins as well as bone and cartilage formation in calcified vascular lesions has suggested that osteogenic mechanisms may play a role in vascular calcification [24]. Interestingly, calpain-2 has been shown to regulate matrix mineralization in a rat growth plate chondrocyte culture model [25], suggesting that calpains could be involved in vascular calcification. Furthermore, it has been suggested that apoptotic bodies derived from vascular smooth muscle cells may act as nucleating structures for calcium crystal formation and thus initiate vascular calcification [26]. A recent paper showed that vascular smooth muscle cell apoptosis in transgenic mice induced features of plaque vulnerability in atherosclerosis [27]. The fact that calpain regulates oxLDL-induced apoptosis [12,28], and possibly other types of vascular cell death, combined with the above-mentioned findings, suggests that this enzyme may be a central regulator of vascular calcification, and play an important role in the development of vulnerable plaques.

## Conclusion

Our results suggest that calpain-1 is commonly active in carotid artery atherosclerotic plaques, and that calpain activity is colocalized with cell death and inversely associated with symptoms.

## Abbreviations

OxLDL: oxidized low-density lipoprotein.

## Competing interests

The authors declare that they have no competing interests.

## Authors' contributions

IG, TCS, ND, LMP, JFF, MPSA, and IPA contributed to the design of the study, LMP and JFF recruited the study participants and collected the samples. IG, MN, TCS, ND, MPSA, and IPA contributed to the collection of data, IG, MN, ND, MPSA, and IPA analysed the data. IG and IPA wrote the draft manuscript, ND and MPSA critically reviewed the manuscript. All authors read and approved the final manuscript.

## Pre-publication history

The pre-publication history for this paper can be accessed here:


